# Editorial: The human neuroscience of music therapy in neurodegenerative diseases

**DOI:** 10.3389/fnhum.2026.1800758

**Published:** 2026-02-19

**Authors:** Veronica Rivi, Takao Yamasaki

**Affiliations:** 1 Department of Life Sciences, University of Modena and Reggio Emilia, Modena, Italy; 2 Department of Neurology, Minkodo Minohara Hospital, Fukuoka, Japan

**Keywords:** human neuroscience, multimodal care, music therapy, neurodegenerative diseases, neuromodulation, predictive processing, sensory integration

Long before the development of neuroimaging, electrophysiology, or formal models of brain function, music was already being used in clinical and rehabilitative contexts in ways that structured movement, emotional expression, and social interaction (Thaut, 2015). What distinguishes the present moment is not the novelty of music as an intervention, but the capacity of human neuroscience to explain, quantify, and integrate its effects into contemporary models of brain function (Scharfen and Memmert, 2024). Advances in systems neuroscience, network analysis, and computational approaches now enable music therapy to be examined as a structured neuromodulatory intervention and neuroscientifically grounded practice with clear relevance for clinical care (Zanchi et al., 2024; Tascedda et al., 2024).

The contributions to *The Human Neuroscience of Music Therapy in Neurodegenerative Diseases* trace a progression from fundamental neural mechanisms, through motor and affective regulation, toward clinical integration and scalable implementation. Read in this way, the Research Topic reflects a field that has moved beyond the question of whether music therapy is effective, and toward the more consequential challenge of how its effects can be systematically explained, optimized, and embedded within multimodal models of care.

At the foundation of this framework lies the role of temporal structure. The nervous system is intrinsically predictive and time-sensitive, relying on the continuous alignment of internal dynamics with external events (Dimakou et al., 2025). Music, as a temporally organized stimulus, engages this predictive architecture with high efficiency (Basiński et al., 2023). Evidence for this emerges from the work of Meng et al., who demonstrate that auditory rhythmic adaptation can influence lower-limb joint mechanics during complex motor tasks. Although conducted outside a neurodegenerative population, the findings illustrate a fundamental principle: rhythmic auditory input can recalibrate motor planning, coordination, and stability by coupling external temporal cues with internal sensorimotor processes. Such mechanisms are not condition-specific; they reflect core properties of human motor control that can be recruited across health and disease.

These insights provide a bridge to the clinical context of Parkinson's disease, where disruptions of timing, automaticity, and basal ganglia function are central features (Grahn, 2009). The systematic review and meta-analysis by Li et al. situate rhythmic and melodic interventions within this clinical framework, demonstrating that neurologic music therapy exerts measurable effects across both motor and non-motor domains. Rhythmic auditory stimulation supports gait and balance through preserved auditory–motor pathways, while melodic and harmonic elements are thought to influence motivation, cognition, and mood via frontostriatal and limbic circuitry. Importantly, these findings support the view that music-based interventions may operate synergistically with pharmacological treatments by harnessing preserved timing, reward, and sensorimotor pathways in the aging brain, reinforcing the need for interdisciplinary collaboration between neurologists, therapists, and music therapists (Rivi et al., 2021; Blom et al., 2023).

The capacity of music to regulate neural function, however, extends beyond motor systems. Affective and autonomic regulation emerge as equally important dimensions. Shepherd et al.'s electrophysiological investigation of a purpose-designed song intended to reduce subclinical anxiety illustrates how specific musical structures can be linked to measurable changes in neural activity. By examining electrophysiological correlates rather than relying solely on subjective outcomes, the study exemplifies a shift toward neurophysiological accountability in music therapy research. Musical parameters are no longer treated as interchangeable features, but as variables that can be systematically manipulated to target defined neural processes.

This emphasis on specificity resonates with broader efforts to align music-based interventions with pharmacological approaches that also modulate arousal, anxiety, and mood. As music therapy becomes more precisely characterized, opportunities emerge for integrated treatment strategies in which auditory stimulation and medication act on complementary neural pathways. Such strategies may reduce side effects, enhance adherence, or extend therapeutic benefit, particularly in populations where long-term pharmacological burden is a concern.

In this broader context, music therapy can be conceptualized within a sensory integration framework, in which structured auditory stimulation contributes to the coordinated regulation of affective, cognitive, and autonomic processes. Such approaches have been proposed as clinically relevant, non-pharmacological strategies for promoting healthy aging and supporting dementia management, particularly in populations where long-term pharmacological burden is a concern (Maneemai et al., 2024).

The relevance of these mechanisms across diagnostic boundaries becomes evident in the meta-analysis by Navarro et al., examining music interventions in autism spectrum disorder. Although autism spectrum disorder is not neurodegenerative in nature, it shares key alterations in predictive processing, sensory integration, and network connectivity with neurodegenerative conditions (Cole et al., 2025; Yamasaki et al., 2017). Music's structured temporal and emotional cues provide a scaffold for social engagement, joint attention, and affect regulation, supporting the view that music therapy operates on transdiagnostic neural mechanisms. This perspective aligns with emerging dimensional models of brain function and suggests that clinical deployment may be most effective when organized around shared functional targets rather than categorical diagnoses.

As understanding of how music engages neural systems continues to mature, a central challenge comes into focus: how such knowledge can be translated into interventions that are both clinically robust and viable at scale. This challenge shifts attention from isolated therapeutic effects to questions of delivery, continuity, and ecological validity. In this context, Yu et al.'s scoping review of digital music-based interventions for individuals with acquired brain injury maps a rapidly expanding technological landscape characterized by innovation alongside considerable heterogeneity. While digital platforms offer unprecedented opportunities for personalization and access, their clinical impact ultimately depends on the extent to which they are guided by coherent neuroscientific principles rather than *ad hoc* design choices.

The RadioMe system described by Street et al. illustrates how this translation can be realized in practice. By embedding music within an adaptive, home-based environment responsive to user behavior and physiological state, RadioMe reframes music therapy as a continuous regulatory resource rather than a discrete clinical event. For individuals living with chronic neurological conditions, such systems offer a pathway for extending care beyond the clinic, supporting adherence, and potentially reducing reliance on pharmacological escalation through enhanced self-regulation and engagement.

Across this Topic, a unifying principle becomes clear. The clinical impact of music therapy is maximized when it is integrated within multimodal therapeutic ecosystems that include pharmacology, physical rehabilitation, cognitive therapy, and digital health infrastructures. This integrated perspective raises important questions for future research and practice, including how music-based interventions should be timed relative to medication cycles, how they may interact with pharmacodynamics, and what institutional frameworks are required to embed them within standard care pathways.

**Figure 1:**
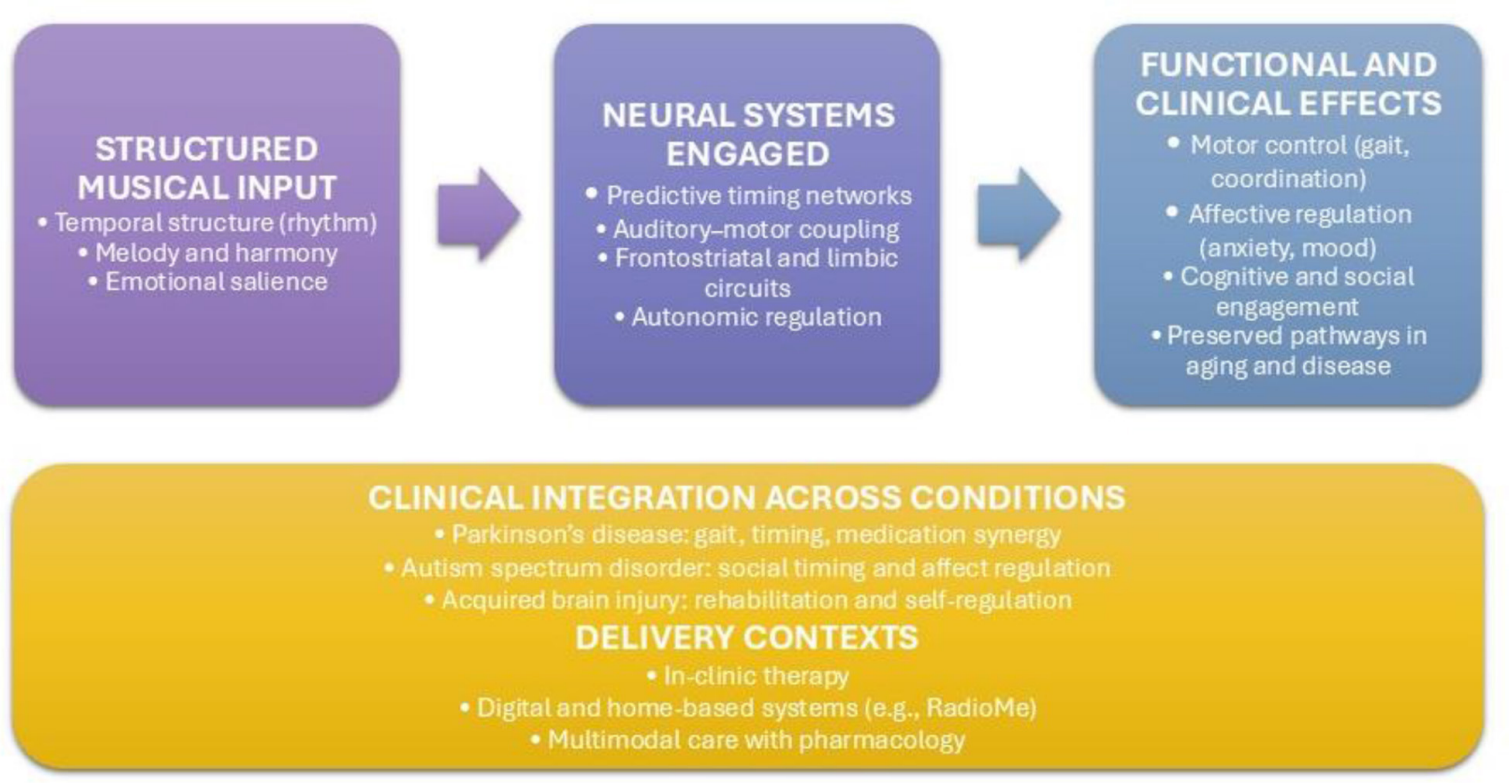
Music therapy as a predictive and integrative intervention within human neuroscience and clinical care.

Taken together, this Research Topic reflects a field that has reached a new level of human neuroscience (Figure 1), one that treats the brain not only as a biological organ, but as a dynamic, predictive system shaped by structured sound, embodied action, and clinical context.

Structured musical input, characterized by temporal organization and emotional salience, engages distributed neural systems involved in predictive timing, auditory–motor coupling, affective processing, and autonomic regulation. These neural engagements support functional outcomes across motor, affective, cognitive, and social domains, including the recruitment of preserved pathways in aging and neurological disease. When embedded within clinical contexts—such as Parkinson's disease, autism spectrum disorder, and acquired brain injury—and delivered through in-clinic and digital systems, music therapy functions as an integrative, neuroscience-informed component of multimodal care alongside pharmacological treatment.
